# A psychometric investigation of the multiple-choice version of Animated Triangles Task to measure Theory of Mind in adolescence

**DOI:** 10.1371/journal.pone.0264319

**Published:** 2022-03-10

**Authors:** Naja Kirstine Andersen, Martin Køster Rimvall, Pia Jeppesen, Mette Bentz, Jens Richardt Møllegaard Jepsen, Lars Clemmensen, Rikke Kart Jacobsen, Else Marie Olsen

**Affiliations:** 1 Child and Adolescent Mental Health Centre, Mental Health Services, The Capital Region, Hellerup, Denmark; 2 Department of Child and Adolescent Psychiatry, Copenhagen University Hospital – Psychiatry Region Zealand, Roskilde, Denmark; 3 Department of Clinical Medicine, Faculty of Health and Medical Sciences, University of Copenhagen, Copenhagen, Denmark; 4 Center for Neuropsychiatric Schizophrenia Research and Center for Clinical Intervention and Neuropsychiatric Schizophrenia Research, Mental Health Services, The Capital Region, Hellerup, Denmark; 5 Copenhagen Research Center for Mental Health, CORE, Mental Health Center Copenhagen, Copenhagen University Hospital, Copenhagen, Denmark; 6 Center for Clinical Research and Prevention, Bispebjerg and Frederiksberg Hospital, Copenhagen, The Capital Region, Denmark; 7 Department of Public Health, Faculty of Health and Medical Sciences, University of Copenhagen, Copenhagen, Denmark; 8 Psychiatric Center Ballerup, Mental Health Services, Capital Region of Denmark, Ballerup, Denmark; University College London, UNITED KINGDOM

## Abstract

The Animated Triangles Task (AT) is commonly used to measure Theory of Mind (ToM). AT can be scored by clinicians based on participants’ verbal responses (AT-verbal) or using a multiple-choice paradigm (AT-MCQ). This study aimed to evaluate the validity of the less time-consuming AT-MCQ. To do this, we examined agreement and correlations between the AT-MCQ and the original AT-verbal scores in 1546 adolescents from a population-based sample. As a supplementary analysis of known-groups validity, we examined if AT-MCQ was as sensitive as AT-verbal in detecting ToM-limitations in 54 adolescents with autism spectrum disorder (ASD), using register-data. The agreement between AT-verbal and AT-MCQ varied markedly across test items. Scores on the two scoring methods were weakly correlated. Both scoring methods weakly detected differences between adolescents with and without ASD in this population-based sample. Most participants had appropriate responses on both AT-MCQ and AT-verbal, which yielded overall acceptable agreement. However, the feasibility of using either scoring methods to measure ToM-limitations in adolescents from the general population is questionable.

## Introduction

Theory of Mind (ToM) refers to a person’s ability to attribute mental states such as intentions, beliefs and dispositions to others [1]. Several psychiatric disorders, including autism spectrum disorders (ASD) and schizophrenia, are characterized by deficits in ToM [2, 3], and this has shown to be an particular important predictor of social functioning in some patient groups [4]. Investigation of ToM in individuals from the general population is important to understand the impact and nature of ToM impairments in the development of psychiatric disorders.

Numerous measures of ToM exist, but the assessment of ToM is challenged by the fact that many instruments show ceiling effects [5] and are influenced by factors other than ToM, such as neurocognitive functioning [6]. Moreover, some individuals (e.g. individuals with high-functioning ASD) might do well in laboratory tests based on social cues, even though they have real-life ToM-difficulties [7].

The Animated Triangles Task (AT) (also known as the Frith-Happé Animations) was developed by Abell, Happé, & Frith [8] to meet these limitations of traditional ToM tests. AT consists of a series of computer-based animations, to which the participants are asked to describe the actions of two triangles moving around the screen. The animations are non-verbal and present geometric shapes as stimuli which make the ToM attribution exclusively dependent on movement, and not on verbal or facial cues [8]. Moreover, AT accounts for non-mentalizing performance differences as it includes control conditions that do not demand ToM abilities. Due to these advantages, the AT is one of the most widely used ToM tests [9].

The original version of AT (AT-verbal) is based on the verbal responses of the test-person, and can be rated according to the scoring method outlined in Abell et al. [8] or Castelli et al. [10]. The analysis and scoring of AT are rather time consuming and somewhat subjective, as trained clinicians are required to interpret and score AT based on the verbal responses of the test-person [11]. For these reasons White, Coniston, Rogers, & Frith [11] developed an ‘objective’ scoring procedure for AT with multiple-choice questions (AT-MCQ). In AT-MCQ, the participants categorize the animations as one of three types. Additionally, after certain animations, the participants are asked to select the feeling, among five options, that best describes the mental state of each triangle.

The multiple-choice format makes AT-MCQ simple and quick to administer and requires minimal professional training. In addition to the advantages regarding time consumption, AT-MCQ does not rely as heavily on the participant’s verbal capacity and reduce the risk of rating bias. Also, AT-MCQ does not require national scoring guides and the animations are thought to be more culturally neutral than tasks depicting humans in social situations [12]. Hence, the AT-MCQ scoring method is potentially more suitable for international and cross-cultural studies than AT-verbal, where performance has shown to be highly influenced by nationality [13]. Furthermore, the multiple-choice format has made White et al.’s [11] AT-MCQ suitable for imaging studies examining neural activity related to ToM [14–16] as well as internet-based research [17].

In the initial study introducing AT-MCQ, White et al. [11] showed in a study including 16 adults with ASD and 15 typically developing adults that AT-MCQ, as well as the original ‘subjective’ method, was able to detect significant differences in performance on AT between the two groups. The ASD group was significantly worse at categorizing the three types of animations in AT-MCQ (*g* = -1.33 [-2.11; -0.56]) and less often selected the correct mental states of the triangles (*g* = -1.82 [-2.66; -0.98]). Hence both conditions in AT-MCQ were able to discriminate between the ASD group and the healthy control group, indicating initial validity of the AT-MCQ.

A recent meta-analysis by Wilson [9] tested whether individuals with ASD showed ToM impairments in the AT task (using any scoring method) in an aggregated sample of 3099 children and adults. The meta-analysis found that individuals with ASD performed significantly worse than individuals without ASD, however, the group differences were small, and group differences could be observed not only in the ToM condition but also in the control conditions. The meta-analysis did not aim at comparing the scoring methods, but the author observed a trend towards smaller group differences between ASD and non-ASD individuals in the AT-MCQ compared to the AT-verbal.

To our knowledge, only one previous study has examined the validity of AT-MCQ compared to AT-verbal. Livingston et al. [18] conducted a web-based version of AT and examined the associations between one domain of AT-MCQ and AT-verbal scores in 285 healthy adults, and found statistically significant associations of r_s_ = 0.26–0.48. The authors conclude that their study provides further evidence for the validity of the quick and more objective AT test, AT-MCQ.

The primary aim of the current study was to further examine the psychometric properties of AT-MCQ by examining the agreement between AT-MCQ and AT-verbal in a large sample of adolescents from the general population. We hypothesize that the agreement will be at least moderate according to the Landis and Koch guidelines [19]. As a secondary analysis, we examined if AT-MCQ is as sensitive as AT-verbal in detecting expected differences in ToM between individuals with and without ASD, as a measure of known-groups validity. We hypothesize that both AT-MCQ and AT-verbal significantly discriminate between the groups.

## Method

### Participants

The study was conducted as part of the 16-year follow-up of the Copenhagen Child Cohort 2000 (CCC2000). The CCC2000 is a longitudinal cohort study with the overall aim to broadly investigate indicators of mental health problems in a developmental perspective, by following a general birth cohort of 6090 children born in the County of Copenhagen in the year 2000 [20].

**Fig 1:**
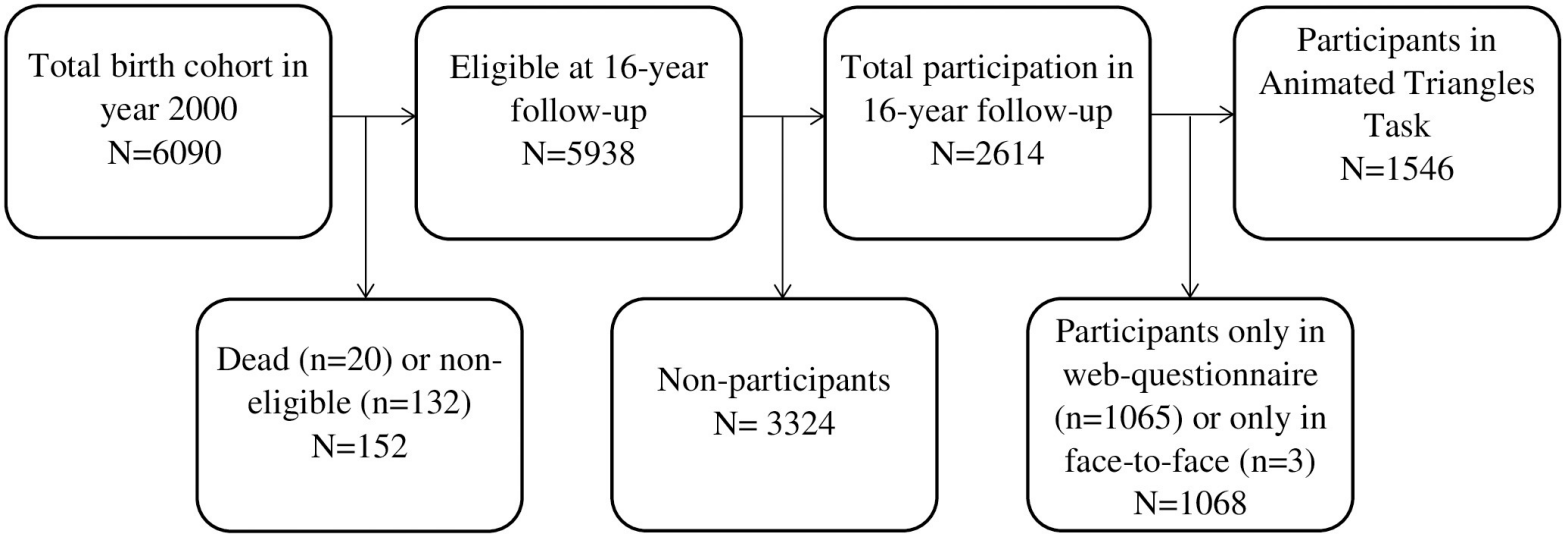
Flowchart of participation in the Animated Triangles Task.

The 16-year follow-up consisted of a web-questionnaire and a subsequent face-to-face examination both covering a wide range of mental and physical health items. A total of 5938 of the adolescents were eligible for this follow-up [20]. Of these, 1546 completed the Animated Triangles Task as part of the face-to-face examination (see Fig 1). Of the participants in the 16-year follow-up, 54 had been diagnosed with an ASD diagnosis according to the Danish patient registry.

### Assessment instruments

#### Animated Triangles Task

The Animated Triangles Task (AT) consists of 12 short animations (34–45 sec) with 4 random, 4 goal-directed (GD) and 4 ToM animations presented in a pseudo-randomized order [8]. The animations depict a big red and a small blue triangle moving around the screen. In the random animations there is no interaction between the triangles, in the GD animations there is a physical interaction between the triangles (e.g. chasing) and in the ToM-animations one triangle is acting with the intention to influence the mental state of the other triangle (e.g. coaxing).

The present study combined the test procedure for AT-MCQ and AT-verbal as outlined in White et al. [11]. The participants were instructed to give a verbal description of the actions or the plot in the animations and then categorize each animation as one of the three types:

- **Type 1**: No interaction. There is no obvious interaction between the triangles and their movement appears random. (Random animations).
- **Type 2**: Physical interaction. An interaction between the triangles in which actions are directed toward each other in order to achieve specific goals. (Goal-directed (GD) animations).
- **Type 3**: Mental interaction. An interaction between the triangles involving the manipulation of the emotions and thoughts of one triangle by the other. (ToM animations).

The above definitions from White et al. [11] were in a previous study [21] translated into Danish and back-translated in agreement with the authors. The validity of the Danish version of AT concerning the ability to discriminate between high-functioning ASD and typically developing children and adolescents has been shown by Clemmensen et al. [22]. For the study CCC2000, the wording of the above definitions was slightly simplified to make them suitable for a younger sample. The definitions in written form were available to the participants during the whole testing. The participants were also instructed that they would receive extra questions following some of the animations.

Prior to testing the participants were presented with three practice animations (one random, one GD and one ToM animation) and feedback was given on the participant’s response. An explanation of the plot was given if the categorization of the animation was wrong. The 12 test animations were presented whether the participants answered correctly in the practice animations or not. If participants did not begin verbal description while watching the test animations, the tester prompted the participants by saying “Please tell me what happens in this animation”.

Following each of the four ToM animations, the participant was given two supplementary questions regarding the feelings of the small and the big triangle: “How do you think, the small triangle feels at the end of the animation?” and “How do you think the big triangle feels at the end of the animation?”. The participant was asked to choose from five potential feelings presented in writing and read aloud. The participants received the feelings questions regardless of their categorization of animation type.

Categorization of the type of animation and categorization of feelings were registered by the tester. The verbal responses were recorded and transcribed and subsequently rated by two clinical psychologists (authors LC and MB) and one trained psychology student (author NKA).

#### Rating

White et al.’s [11] AT-MCQ is based on the responses to the multiple-choice questions (MCQ). It involves the number of correct categorizations of animations on a scale from 0–12 (MCQ-categorization) and number of correctly classified feelings for the small and big triangle in the ToM animations on a scale from 0–8 (MCQ-Feeling). In the current study, AT-MCQ-categorization scores are further analyzed separately for random, GD and ToM animations on a scale from 0–4.

AT-verbal is based on the transcript of the verbal responses of the actions in the animations. In the current study, we applied two of four AT-verbal scales outlined by Castelli et al. [10]; Appropriateness and Intentionality. These scales are the most frequently used AT-verbal scales and are the scaled used in White et al.’s [11] development of AT-MCQ. We scored the verbal responses according to Appropriateness on a scale from 0–3 and Intentionality on a scale from 0–5 for each animation. Scores are summed to an Appropriateness score (0–12) and an Intentionality score (0–20) for each animation type. A high Appropriateness score reflects an accurate understanding of the plot of the animation. A high Intentionality score reflects a high level of mental state attribution and use of elaborated mental state language (e.g. 0: “they just move around” or 5: “the big triangle tries to persuade the small triangle”). A high Appropriateness score is desirable in all animations, whereas the desired level of Intentionality score is dependent on the animation type: a low score is appropriate for random animations, a moderate score is appropriate for GD animations and a high score is appropriate for ToM animations.

In the analyses of agreement between AT-MCQ and AT-verbal, we chose to group AT-verbal intentionality scores in three groups (grouped Intentionality score) to match the three types of animations in the MCQ-categorization. The grouping was conducted by carefully matching the description of the AT-MCQ animation type with the description of the individual AT-verbal Intentionality scores, and they were matched as follows:

- Type 1 (‘no interaction’) corresponds to a low Intentionality score of 0 or 1 (‘action, nondeliberate’ or ‘deliberate action with no other’)
- Type 2 (‘physical interaction’) corresponds to a moderate Intentionality score of 2 or 3 (‘deliberate action with another’ or ‘deliberate action in response to other’s action’)
- Type 3 (‘mental interaction’) corresponds to a high Intentionality score of 4 or 5 (‘deliberate action in response to other’s mental state’ and ‘deliberate action with goal of affecting other’s mental state’)

### Variables for external validation

#### Autism spectrum disorders

To determine the presence of autism spectrum disorders, the cohort was linked to the Danish National Patient Registry [23], which contains information on psychiatric diagnoses ascribed to all in and out-patients hospitalized in Denmark since 1995, using the International Classification of Diseases 10^th^ revision (ICD-10). In Denmark there is free and equal access to healthcare services, including child mental health, and hospital diagnoses are rendered by medical doctors following diagnostic procedures. The Danish National Patient Registry has been found to have good coverage and validity concerning major psychiatric disorders, including ASD [24, 25]. For this study, we obtained F84 ICD-10 diagnoses given from birth of the participant until June 2017.

#### Self-rated difficulties

Self-rated difficulties was examined using ‘The Strengths and Difficulties Questionnaire’ (SDQ) for 11-17-year-olds [26]. SDQ consists of 25 questions regarding 1) emotional symptoms, 2) conduct problems, 3) hyperactivity/inattention, 4) peer relationship problems and 5) prosocial behavior. For the current study, we used the scale Total Difficulties consisting of scores of domain 1–4 added together on a scale ranging from 0–40 (higher scores reflect more psychopathology). SDQ is a widely used and validated instrument for self-evaluation and screening of mental health problems in community samples of adolescents [27], and the SDQ has proved useful and valid in the Danish population [28]. In the current study, we found a Cronbach’s alpha = 0.63.

#### Intelligence

A proxy measure of intelligence was estimated from the non-verbal subtest ‘Block design’ (BD) in ‘Wechsler Intelligence Scale for Children 3. version’ (WISC-III) that was applied in the 11-year follow-up. No IQ-measure at age 16 was administered, and therefore we applied the BD-score at age 11 as a proxy measure of current intelligence. Although IQ was measured some years prior to the ToM measures, overall IQ-status is considered a constitutional and fairly stable trait over ages [29]. The BD-test score is highly correlated with IQ-score (.73) [30, 31]. In BD the participants use colored blocks to recreate a presented pattern as quickly as possible. BD-scores are reported in mean age-adjusted scale scores from 1–19. The performance in BD at age 11 was included as a potential confounder in a sensitivity analyses of a subsample, as BD-score was available only in 893 out of 1546 participants in the current study.

### Ethics

The CCC2000 study was approved by the Danish Data Protection Agency (CSU-FCFS-2016-004, I-Suite 04544), and the Committee on Health Research Ethics in the Capital Region of Denmark has evaluated the project (protocol 16023242). The Helsinki Declaration regarding the use and anonymization of personal data was followed. The data is only to be used for research purposes, and participants gave written informed consent.

### Statistical analysis

The statistical analyses were conducted using Stata 15. Characteristics of participants compared to non-participants in the 16-year follow up were examined with Pearson’s chi-squared test.

Inter-rater agreement for the AT-verbal scoring was calculated for 246 subjects (approx. 16% of the full sample) using the Intraclass Correlation Coefficient (ICC). Internal consistency of the AT-MCQ and AT-verbal, including evaluations of specific items, was calculated using Cronbach’s alpha (α).

Percentage agreement between MCQ-categorization scores and AT-verbal grouped Intentionality scores were examined using the inter-rater coefficients Gwet’s AC [32] and Cohens Kappa.

Sensitivity, specificity, positive predictive value and negative predictive value were calculated for the MCQ-categorization scores with the grouped Intentionality scores as reference to examine the ability of AT-MCQ to obtain the same scores as the original AT-verbal. As there are three response categories, these calculations were made with constructed binary variables with the ‘correct’ score as ‘positive’ and the remaining two scores as ‘negative’. Association between AT-MCQ and AT-verbal were calculated using Spearman’s rank correlation. Nonparametric analyses are used due to skewness of the AT-scores (ranging from 0.3 to 2.1). See information on skewness and kurtosis in S6 Table. We examined differences in AT-scores between boys and girls to examine potential bias of sex, using Mann-Whitney. We examined the effects of IQ on AT scores using Spearman Rank Correlation and Analysis of variance.

Differences in AT performance between participants with and without ASD were examined by Wilcoxon Rank Sum Test (Mann-Whitney) and effect sizes are reported by Hedges’ g (when large differences in sample size) and Cohen’s d (when equal sample size). We further compared the AT-scores for the ASD and non-ASD group for girls and boys separately and for subjects with high and low block-design score separately (dichotomized at the 50^th^ percentile) using Mann-Whitney. Effect sizes between AT-MCQ scores for ASD vs. non-ASD groups in previous studies are reported by Hedges’ g [33].

#### Missing data

There were no missing data in AT-MCQ, as AT-MCQ was conducted in a forced-choice format. Missing data occurred in AT-verbal scores due to errors in recording, transcription or due to lack of verbal responses from the participant. For Intentionality scores, responses without any description of movement are also considered missing data, as Intentionality cannot be scored.

In analyses of AT-verbal, sum scores for animation type we imputed the mean from the remaining scores in the specific animation type if a subject had two or fewer missing scores. If a subject had more than two missing scores within the same animation type, the subject was dropped from the analysis of that specific animation type. Following imputation, there was no missing Appropriateness scores, and between 5 and 16 missing Intentionality scores across the three animation types.

## Results

### Characteristics of participants

Significantly fewer boys participated in the 16-year follow-up compared to non-participants (members of the birth-cohort not participating in the follow-up) (see Table 1). Also, the participants less often had autism spectrum disorders compared to non-participants, although this difference was nonsignificant.

**Table 1:** Characteristics of the participants in the CCC2000 16-year follow-up compared to non-participants.

Variable	Entire Cohort Alive (N = 6070)	Participants (N = 1546)	Non-participants (N = 4524)	Participants vs. non-participants
	N (%)	N (%)	N (%)	P-value
Sex, boys	3115 (51%)	695 (45%)	2420 (54%)	<0.001
Autism spectrum disorder^a^	259 (4%)	54 (4%)	205 (5%)	0.081

^a^ Participants having an ICD-10 F84 diagnosis.

### Inter-rater reliability

Inter-rater reliability for AT-verbal Appropriateness and Intentionality scores was excellent for all animations (ICC = 0.77 to 0.97) (See S1 Table).

### Internal consistency

The internal consistency of the AT-MCQ was α = 0.32 for random animations, α = 0.21 for GD animations, and α = 0.36 for ToM animations. The internal consistency of AT-verbal Appropriateness scores was α = 0.57 for random animations, α = 0.33 for GD animations, and α = 0.54 for ToM animations, and for AT-verbal Intentionality scores, α = 0.52 for random animations, α = 0.30 for GD animations, and α = 0.51 for ToM animations. Subsequent analyses showed that the Cronbach’s a coefficient would increase from 0.21 to 0.28 if animation 3 was dropped from the GD animations in AT-MCQ, and increase from 0.51 to 0.55 if animation 2 was dropped from the analysis of Intentionality in ToM animations.

### Distribution of scores

The distribution of MCQ-categorization and AT-verbal grouped Intentionality scores for each animation is presented in Table 2. Overall, the vast majority chose the correct animation type and obtained the appropriate intentionality score in both random, GD and ToM animations. However, fewer participants obtained the appropriate high intentionality score in ToM animations and fewer correctly identified animation 3 as a GD animation.

**Table 2:** Distribution of AT-MCQ categorizations and AT-verbal intentionality scores given in percentages.

	AT-MCQ categorization			AT-verbal grouped Intentionality score		
	Type 1	Type 2	Type 3	1 (score 0–1)	2 (score 2–3)	3 (score 4–5)
*Random*						
Animation 4	92%	8%	1%	91%	8%	<1%
Animation 7	98%	*	*	97%	3%	<1%
Animation 9	94%	5%	1%	95%	5%	<1%
Animation 12	85%	14%	<1%	78%	21%	<1%
*Goal-directed*						
Animation 1	4%	88%	8%	4%	96%	<1%
Animation 3	1%	32%	67%	<1%	87%	12%
Animation 6	3%	70%	27%	3%	90%	7%
Animation 10	1%	89%	10%	1%	97%	2%
*ToM*						
Animation 2	<1%	3%	97%	1%	66%	33%
Animation 5	1%	26%	73%	<1%	51%	48%
Animation 8	1%	6%	93%	<1%	49%	51%
Animation 11	1%	4%	95%	2%	38%	60%

*too few subjects.

Abbreviations: AT-MCQ: Animated Triangles Task—multiple choice questions; AT-verbal: Animated Triangles Task—verbal response; ToM: Theory of Mind.

### Agreement between AT-MCQ and AT-verbal

The percentage agreement between MCQ-categorization and grouped Intentionality scores was highest for random animations and ranged between 83% and 96% (see Table 3). The agreement for GD animations ranged between 39% and 88%, with animation 3 having a markedly lower agreement than the other GD animations. The agreement was lowest for ToM animations, where the agreement ranged between 35% and 61%, with animation 2 having the lowest agreement. Due to skewness of the distribution of scores where the vast majority of responses fall in one category, the Kappa coefficients are low in all animations (0.01–0.28) despite high percentage agreement. The Gwet’s AC statistics, that to some extend overcome the paradox of high agreement and low reliability observed with the Cohen’s Kappa coefficients [32], on the contrary show values more similar to the percentage agreement (0.15–0.96). The Gwet’s AC coefficients corresponded to at least moderate agreement [19] in all but three animations (Animations 2, 3 and 8).

**Table 3:** Agreement between AT-MCQ categorizations and AT-verbal grouped intentionality scores.

	Percentage agreement	Gwet’s AC	Cohen’s Kappa
*Random animations*			
Animation 4	87%	0.86	0.17
Animation 7	96%	0.96	0.13
Animation 9	93%	0.92	0.28
Animation 12	83%	0.80	0.43
*Goal-Directed animations*			
Animation 1	85%	0.84	0.04
Animation 3	39%	0.20	0.05
Animation 6	74%	0.68	0.25
Animation 10	88%	0.87	0.07
*Theory of Mind animations*			
Animation 2	35%	0.15	0.01
Animation 5	59%	0.46	0.20
Animation 8	51%	0.39	0.02
Animation 11	61%	0.53	0.07

Abbreviations: AT-MCQ: Animated Triangles Task—multiple choice questions; AT-verbal: Animated Triangles Task—verbal response.

Sensitivity was generally high for the correct score in all animations (74.5 to 97.7%) (see Table 4). That is, MCQ-categorization score was likely to be associated with the corresponding grouped Intentionality score. Only Animation 3, a GD animation, has low sensitivity (33.7%) as most participants wrongly categorize it as a ToM animation. Specificity varies markedly but is generally low (3.2 to 69.6%). PPV is high in random and GD animations (86.1 to 97.9%), but lower in ToM animations (33.5 to 61.4%). NPV is fairly high in ToM animations (59.2 to 72.3%), but lower in random and GD animations (6.0 to 66.5%).

**Table 4:** Sensitivity, specificity, positive predictive value (PPV) and negative predictive value (NPV) for the correct AT-MCQ categorization with the corresponding grouped AT-verbal intentionality score as reference method^a^.

	Sensitivity	Specificity	PPV	NPV
*Random animations*				
Animation 4	93.3	26.5	93.1	27.1
Animation 7	98.1	13.6	97.5	17.1
Animation 9	95.9	35.4	96.5	31.8
Animation 12	93.7	45.3	86.1	66.5
*Goal-Directed animations*				
Animation 1	88.3	17.2	96.1	6.0
Animation 3	33.8	78.5	91.5	14.6
Animation 6	74.3	69.6	95.5	23.7
Animation 10	89.6	25.6	97.9	6.0
*Theory of Mind animations*				
Animation 2	97.3	3.2	33.5	70.2
Animation 5	84.6	36.9	55.2	72.3
Animation 8	94.6	8.0	51.2	59.2
Animation 11	97.7	8.3	61.4	70.8

^a^The corresponding AT-verbal score is 0–1 for random animations, 2–3 for goal-directed animations and 4–5 for Theory of Mind animations.

Abbreviations: AT-MCQ: Animated Triangles Task—multiple choice questions; AT-verbal: Animated Triangles Task—verbal response.

As shown in Table 5, the AT-MCQ scales, MCQ-categorization, MCQ-categorization for ToM animations and MCQ-feelings, and the AT-verbal Appropriateness and Intentionality scales for ToM animations were significantly and positively correlated to each other, but the associations were weak.

**Table 5:** Correlations between AT-MCQ scores and AT-verbal scores in ToM animations.

	*AT-MCQ*		
	MCQ-categorization (0–12)	MCQ-categorization ToM (0–4)	MCQ-feeling (0–8)
*AT-verbal*			
Appropriateness ToM (0–12)	0.1024*	0.1408*	0.1893*
Intentionality ToM (0–20)	0.1433*	0.2046*	0.1391*

* p < .05.

Abbreviations: AT-MCQ: Animated Triangles Task—multiple choice questions; AT-verbal: Animated Triangles Task—verbal response; ToM: Theory of Mind.

### Effects of sex and IQ

Comparisons of AT-scores between boys and girls showed that boys and girls performed equally good in AT-MCQ. In AT-verbal, boys had significantly higher Intentionality scores in ToM and GD animations, and higher Appropriateness scores in ToM animations (see S2 Table). However, again, the observed differences in mean scores were small. There were no significant differences in the remaining conditions.

The IQ proxy measure Block Design measured at age 11 correlated with AT scores. The AT-MCQ variables MCQ-feelings and MCQ-categorization and all AT-verbal Appropriateness scores were positively and statistically significantly correlated to Block Design scores with spearman correlation coefficients ranging from r_s_ = 0.104 to 0.172. Regarding AT verbal Intentionality scores, there was a positive and statistically significant correlation to Block Design scores in ToM animations (r_s_ = 0.145), no significant correlation in goal-directed animations (r_s_ = 0.042) and a negatively and statistically significant correlation in random animations (r_s_ = -0.157). Furthermore, as seen in S3 Table, the individuals with the lowest BD scale score at age 11 generally performed worse in AT, however, the observed differences in mean score were minimal.

### Between-group comparisons

In the AT-MCQ, the mean MCQ-feelings score, but not the mean MCQ-categorization score, were significantly lower for the ASD group than the non-ASD group (see Table 6). For MCQ-categorization separated in animation type, the ASD group performed significantly worse in random animations, but not in ToM or GD animations, compared to the non-ASD group. For AT-verbal, only the Intentionality and Appropriateness scores in GD animations, but not in random or ToM animations, were significantly lower in the ASD group. The observed differences in mean scores for all scales showed small to moderate effect sizes. The ASD group compared to the participants without ASD had significantly more difficulties measured with the ‘Strengths and Difficulties Questionnaire’ (SDQ) (Non-ASD: 9.46 (5.57) vs. ASD: 12.87 (7.22), p = 0.001, *d* = 0.53 [0.43;0.63]).

**Table 6:** Comparison of animated triangles scores between adolescents with and without diagnoses of autism spectrum disorder (ASD).

	Non-ASD n = 1492	ASD n = 54		
	Mean (SD)	Mean (SD)	p-value	Hedges’s g (95% CI)
**AT-MCQ**				
MCQ-categorization (0–12)	10.07 (1.37)	9.98 (1.27)	0.406	0.07 (-0.21;0.34)
-Theory of Mind animations (0–4)	3.59 (0.66)	3.63 (0.56)	0.856	-0.07 (-0.34;0.21)
-Goal-directed animations (0–4)	2.80 (0.84)	2.85 (0.79)	0.556	-0.08 (-0.35;0.20)
-Random animations (0–4)	3.70 (0.60)	3.5 (0.72)	0.013*	0.33 (0.06;0.60)
MCQ-feelings (0–8)	5.33 (1.56)	4.63 (1.96)	0.012*	0.45 (0.18;0.72)
**AT-verbal**				
*Intentionality (0–20)*				
Theory of Mind animations	14.34 (2.54)	13.78 (1.85)	0.199	0.22 (-0.05;0.49)
Goal-directed animations	9.67 (1.52)	9.19 (1.54)	0.029*	0.38 (0.47;0.59)
Random animations	1.97 (1.79)	2.25 (1.85)	0.241	-0.15 (-0.43;0.12)
*Appropriateness (0–12)*				
Theory of Mind animations	6.84 (1.80)	6.72 (2.03)	0.755	0.06 (-0.21;0.34)
Goal-directed animations	9.21 (1.45)	8.69 (1.67)	0.030*	0.36 (0.09;0.63)
Random animations	10.07 (1.95)	9.30 (2.74)	0.067	0.39 (0.12;0.66)

* p < .05.

Abbreviations: AT-MCQ: Animated Triangles Task—multiple choice questions; AT-verbal: Animated Triangles Task—verbal response; ToM: Theory of Mind; ASD: autism spectrum disorders; SD: standard deviation.

### Effect of sex and IQ in the between-group comparisons

To examine the possible effect of sex within the analyses comparing AT-scores in the ASD and non-ASD group, we did stratified analyses. AT scores for boys and girls are shown in S4 Table. This was done separately for individuals with and without ASD. There were no statistically significant differences in mean scores for MCQ-categorization and MCQ-feelings between boys and girls for the non-ASD group or ASD group. AT-verbal scores differed somewhat between boys and girls in the non-ASD group: the boys had significantly higher mean sum Intentionality scores in ToM and GD animations and higher mean Appropriateness scores in ToM animations compared to girls, however, the difference was small.

For the participants who had completed the IQ proxy measure Block Design at age 11 (n = 893), the ASD sample had a slightly higher mean score (M = 10.7, SD = 3.6, n = 23) compared to individuals without ASD (M = 9.9, SD = 3.7, n = 870). We did stratifications for high vs. low IQ (dichotomized at the 50^th^ percentile), as shown in S5 Table. In the non-ASD group the subjects with high IQ had significantly higher mean score on MCQ-categorization and MCQ-feeling than the subjects with low IQ. AT-verbal Intentionality scores were higher for the high IQ subjects in ToM animations and lower in the random-animations compared to low IQ subjects. Appropriateness scores were higher in all conditions for the high IQ subjects in the non-ASD group.

In the ASD group, the subjects with estimated high IQ had significantly higher mean sum Appropriateness scores in GD animations than the subjects with low IQ, but the groups did not differ in any other conditions.

## Discussion

### Main findings

To our knowledge, this is the largest general population sample to be examined by the Animated Triangles Task (AT) with both the AT-MCQ and the AT-verbal scoring methods. We found that the agreement between MCQ-categorization and AT-verbal Intentionality scores varied between the 12 test items, with the lowest agreement in ToM animations. The vast majority of respondents gave appropriate responses using both scoring methods, which resulted in moderate to almost perfect agreement [19] in most animations. Furthermore, we found that AT-MCQ scores (MCQ-categorization and MCQ-feeling) were very weakly associated with AT-verbal scores (Intentionality and Appropriateness scores in ToM animations). Sensitivity and positive predictive value for correct AT-MCQ scores with the corresponding AT-verbal Intentionality score as reference were overall good. Specificity and negative predictive value were generally lower. Neither the AT-MCQ nor the AT-verbal, sufficiently discriminated on group level between participants with and without expected ToM-limitations, as participants with ASD and without ASD overall had similar AT-scores. We found that adolescents with ASD had statistically significantly lower scores in MCQ-feeling, Appropriateness and Intentionality in GD animations, but with only small to moderate effect sizes.

In sum, our study finds overall acceptable agreement between AT-MCQ and AT-verbal scores, but indicates that both scoring methods poorly identify expected ToM-differences between individuals with and without ASD in a general population sample.

### Internal consistency of the AT-MCQ and AT-verbal scales

The internal consistency of both AT-MCQ and AT-verbal was low, which might indicate low relatedness between the individual items (the four animations of each type). The results identified several problematic items within the scoring conditions, meaning that the Cronbach’s alpha coefficient would increase if dropped from the analyses, but no items were problematic across both the AT-MCQ and the AT-verbal scoring methods.

### Agreement between AT-MCQ and AT-verbal

We examined the agreement between the subjects’ AT-verbal Intentionality scores and the MCQ-categorization of the animations as random, GD or ToM, and found overall acceptable agreement. However, the percent agreement ranged between 35% to 96% on the 12 test items. The large variation in agreement demonstrates that the two scoring methods do not correspond to each other in the way we expected. The agreement was markedly lower in Animation 2 (35%) and Animation 3 (39%) compared to the other animations. Especially in Animation 3 the agreement differs from the agreement in the remaining three animations of the same type, which ranged between 70% to 89%. The scoring distribution shows that most adolescents characterized Animation 3 as a ToM type. This indicates that the animation appears to depict more intentional interaction than appropriate for a GD animation and questions the quality of this specific item.

The agreement between AT-MCQ and AT-verbal scores also varied according to animation type; the agreement was high in random animations, generally moderate in GD animations, but relatively low in ToM animations. The lower agreement among ToM animations was a result of a high number of participants receiving only moderate Intentionality scores in the AT-verbal paradigm but being able to correctly categorize the animations as a ToM type in the AT-MCQ paradigm. This means, that the correct interpretation of an animation as a ToM type is not necessarily reflected in advanced ToM attribution in the verbal responses.

Furthermore, it is worth noting that the sensitivity between MCQ-categorization and AT-verbal Intentionality for correct scores are high. This, however, must be seen in relation to the high number of individuals responding correctly. The low specificity shows that AT-MCQ poorly captures the individuals who attribute an inappropriate level of Intentionality and receives an ‘incorrect’ AT-verbal Intentionality score. The analyses of specificity and negative predictive value are made in relatively few subjects as the majority of the adolescence receives the correct AT-MCQ and AT-verbal scores.

In addition to the varying agreement, the low association between AT-MCQ scores (MCQ-categorization, MCQ-feelings) and AT-verbal (Intentionality and Appropriateness scores in ToM animations) suggests that AT results are not constant across scoring methods. Hence, the convergent validity of AT-MCQ, in terms of agreement to AT-verbal, is limited.

Furthermore, AT-MCQ as well as AT-verbal shows signs of ceiling effects in some conditions, suggesting that the scoring methods are unsuitable for detecting subtle ToM-differences in this sample.

### Ability to discriminate between subjects with and without suspected ToM-limitations

We find that both AT-MCQ and AT-verbal show difficulties in detecting ToM variability in a general population sample of adolescents. We expected that participants with ASD would have lower mean scores in Appropriateness and Intentionality in ToM animations and in MCQ-categorization and MCQ-feelings compared to individuals without ASD, but this was overall not the case. We did find some statistically significant differences between participants with and without ASD in the AT-MCQ measure MCQ-feelings and in the AT-verbal Appropriateness and Intentionality scores in GD animations. However, the observed differences in mean scores were very small, and arguable clinically irrelevant, and the statistical difference should be viewed in light the of the large sample size. The effect sizes were *d*<0.50 for all conditions. The inability to detect score differences between participants with and without suspected ToM-limitations might be a reflection of the small variation in both AT-MCQ and AT-verbal scores, as the majority of the participants have correct responses.

We examined the possible effects of IQ and sex by repeating the analyses for boys and girls separately and for individuals with high and low IQ separately. We did not find that sex and IQ affected the AT-scores markedly as the observed mean group differences were very low (see S4 and S5 Tables).

It must be noted that the adolescents with psychiatric disorders who participated in our study are likely relatively well-functioning in spite of their mental disorders, as they agreed to participate in the somewhat demanding assessment at the 16-year follow-up. The insufficiency of AT to discriminate between participants with and without ASD on expected conditions could be due to a relatively high functioning of the participants at the assessment time. This is supported by previous studies using AT in ASD samples showing poorer performance compared to our sample. For example, White et al. [11], showed a mean value of 7.94 (1.95) for the ASD group in MCQ-categorization compared to our mean value of 9.98 (1.27) (*d* = 1.24 [0.73;1.75]). However, in our sample, the between-group comparison showed that the ASD group compared to the participants without ASD had significantly more difficulties measured with the ‘Strengths and Difficulties Questionnaire’, indicating that the ASD group is somewhat affected by psychopathology.

We thus find it likely that our sample of adolescents with ASD do have subtle ToM difficulties that AT was unable to detect. This interpretation is supported by a recent study by Sedgewick et al. [34] that finds qualitative differences in the verbal responses in AT, but no significant differences in AT-verbal scores, between healthy controls and young females with anorexia nervosa and ASD. AT-verbal did not detect the weak central coherence and interpretation bias in the patient group that were identified in the qualitative analysis, even though this might have important clinical implications.

Overall, our study indicates that neither of the two scoring methods of AT convincingly discriminate between adolescents with suspected ToM-deficits and typically developing adolescents in a general population sample. Hence, our study calls for the development of a ToM assessment instrument sensitive enough to detect the broad variation of ToM abilities, including variations in the general population. Future directions to improve sensitivity might involve ToM tests measuring response time as outcome [35]. However, the AT test might still be useful in samples with more pronounced ToM-limitations.

### Comparisons to previous studies using Animated Triangles Task

Metanalytic findings demonstrate that individuals with ASD perform significantly worse in AT-MCQ compared to non-ASD individuals [9]. However, there are indications of the group differences being slightly smaller in AT-MCQ compared to AT-verbal [9]. Additionally, there are some heterogeneity in the findings across studies.

In Kirkovski et al. [16], Fitzpatrick et al. [36], and Young & Brewer [37] there were no significant group differences in either random, GD or ToM animations. Livingston et al. [18] and Brewer et al. [38] found no group differences in random and GD animations and moderate effect size differences in ToM animations (*g* = -0.44 [-0.72;-0.15] and *g* = -0.35 [-0.62;-0.08]), indicating the ability of AT-MCQ to detect ToM limitations in the ASD group. In the studies reporting an aggregated MCQ-categorization score, Clemmensen et al. [22] (*g* = -0.63 [-1.10;-0.16]) found that individuals with ASD generally performed worse than non-ASD individuals, but this was not the case in Moessnang et al. [39] (*g* = -0.13 [-0.28;0.02]).

In the MCQ-feelings category, individuals with ASD had lower scores compared to individuals without ASD in the study by Fitzpatrick et al. [36] and Young & Brewer [37] (*g* = -0.38 [-0.65;-0.11]), but not in Clemmensen et al. [22] (*g* = -0.44 [-0.90;0.03]).

In sum, it varies to what extent and on which conditions AT-MCQ is able to discriminate between subjects with and without ASD. A potential explanation for these inconsistencies may be due to the sample characteristics. Individuals with ASD are not a homogenous group but represent large diversity in social cognition capability as well as in overall functioning. Another explanation regards the psychometric qualities of the test and could be that AT-MCQ is not sensitive enough to detect individual variations in ToM.

### Strengths and limitations

The main strengths of this study include the application of two scoring methods for AT in the same large population-based sample. High inter-rater reliability was found between the raters of the AT-verbal, allowing for reliable comparisons of the two scoring methods in the same sample. Additionally, the access to comprehensive information on psychiatric disorders through the Danish National Patient Registry made it possible to examine AT-scores specifically for individuals with ASD.

The results should also be considered in the light of some important limitations. First, there are several issues pertaining to the measurement of IQ. IQ was not measured at age 16, and the IQ-proxy at age 11 is only measured in a subsample of the participants under study. Furthermore, the IQ-estimate is only based on a sub-test of the WISC, and future studies might advantageously explore more elaborated cognitive characteristics, particularly verbal IQ measures, associated with AT responses, as AT-verbal relies heavily on verbal capabilities. Second, the lack of differentiation between missing Intentionality scores as due to either no description of movement in the verbal responses or due to errors in recording or transcription can also be considered a limitation of the current study. Third, although the CCC2000 sample was overall representative of the general population in Denmark [40], individuals of the CCC2000 who chose to participate in the 16-year-examinations were, as a group, more resourceful. Compared to non-participants, participants less often came from families of lower income and immigrant background, and were less often children of mothers who were single, smokers, younger and less educated, and they less often had parents with a psychiatric disorder [20]. The participants themselves less often had a diagnosed psychiatric disorder compared to non-participants. Hence, due to selection bias, somewhat less variation on the AT scores could also be expected in the current study, compared to the general population. Fourth, the group of adolescents with ASD included individuals diagnosed with an ASD-diagnosis within the period from birth of the participant until June 2017, and the individuals therefore might not be characterized by ASD-symptoms by the time of testing, although ASD generally is considered a stable diagnosis [41]. The autism diagnoses in the Danish registers have generally shown to be valid [24], and the diagnoses reflect real-life clinical practice with regard to diagnosis of ASD. Nonetheless, it could be a limitation of the current study that present autistic traits were not measured. Fifth, in our study, all participants were asked the MCQ-Feelings questions following the ToM animations. This is a deviation from the test procedure introduced by White et al. [11], where participants only receive MCQ-feelings questions if the preceding MCQ-categorization questions is answered correctly. Finally, the insignificant differences in performance in AT between the ASD and non-ASD group may be driven by low power, as the ASD group is fairly small (n = 54). Nonetheless, the differences in mean scores between the groups are minimal.

## Conclusions

AT-MCQ is time efficient, can be done with limited training, is independent of verbal capabilities and can be applied as an online test, which allows for large scale studies. Hence, there are obvious advantages with AT-MCQ compared to AT-verbal. However, our results indicate, that AT-MCQ show ceiling effects and is too easy to apply in a general population sample of adolescents. The vast majority of adolescents easily discriminate between the three types of animations. Also, both the multiple-choice scoring (AT-MCQ) and the original scoring based on verbal responses (AT-verbal) poorly discriminated between adolescence with and without ASD in this population based study-sample of adolescents. Our study calls for further psychometric evaluation of AT, and specifically the AT-MCQ scoring, as our results may not apply to other age groups or clinical populations. The convergence to other ToM assessment instruments and the psychometric properties of AT in other samples should be examined.

## Supporting information

S1 Table Inter-rater reliability for Intentionality and Appropriateness scores on the 12 animations calculated with Inter Class Correlation (ICC). (PDF)

S2 Table Comparison of animated triangles scores between boys and girls. (PDF)

S3 Table Comparison of Animated Triangles scores according to quartiles of scale scores in the IQ-subtest Block Design administered at age 11. (PDF)

S4 Table Comparison of Animated Triangles scores stratified by gender for adolescents with and without autism spectrum disorder (ASD). (PDF)

S5 Table Comparison of Animated Triangles stratified by low and high IQ for adolescents with and without autism spectrum disorder (ASD) reported for subjects with low and high IQ separately. (PDF)

S6 Table Skewness and kurtosis for Animated Triangles scores. (PDF)
