# Supplementary material for: A psychometric investigation of the multiple-choice version of Animated Triangles Task to measure Theory of Mind in adolescence
Source: PLoS One. 2022 Mar 10;17(3):e0264319. doi: 10.1371/journal.pone.0264319 (PMC8912123; doi:10.1371/journal.pone.0264319)
Supplement: S1 Table — (PDF) [file pone.0264319.s001.pdf]

Table S1. Inter-rater reliability for Intentionality and Appropriateness scores on the 12 animations calculated with Inter Class Correlation (ICC).

	**Intentionality**	**Appropriateness**
Item	ICC (CI 95%)	ICC (CI 95%)
Animation 1	0.77 (0.64-0.85)	0.93 (0.90-0.94)
Animation 2	0.91 (0.89-0.93)	0.97 (0.96-0.98)
Animation 3	0.95 (0.94-0.96)	0.96 (0.95-0.97)
Animation 4	0.83 (0.78-0.86)	0.83 (0.79-0.87)
Animation 5	0.96 (0.95-0.97)	0.90 (0.83-0.93)
Animation 6	0.80 (0.75-0.84)	0.95 (0.93-0.96)
Animation 7	0.85 (0.81-0.88)	0.88 (0.85-0.90)
Animation 8	0.90 (0.88-0.92)	0.95 (0.93-0.96)
Animation 9	0.86 (0.83-0.89)	0.87 (0.84-0.90)
Animation 10	0.87 (0.84-0.90)	0.85 (0.75-0.90)
Animation 11	0.92 (0.90-0.94)	0.93 (0.92-0.95)
Animation 12	0.90 (0.87-0.92)	0.90 (0.87-0.92)

CI: 95% confidence interval
