# Supplementary material for: A psychometric investigation of the multiple-choice version of Animated Triangles Task to measure Theory of Mind in adolescence
Source: PLoS One. 2022 Mar 10;17(3):e0264319. doi: 10.1371/journal.pone.0264319 (PMC8912123; doi:10.1371/journal.pone.0264319)
Supplement: S2 Table — (PDF) [file pone.0264319.s002.pdf]

Table S2. Comparison of Animated Triangles scores between boys and girls.

	**Boys**<br>**n=695**	**Girls**<br>**n=851**		
	**Mean (SD)**	**Mean (SD)**	**p-value**	**Cohen's d (95% CI)**
**AT-MCQ**				
MCQ-categorization (0-12)	10.08 (1.36)	10.06 (1.36)	0.825	-0.02 (-0.11;0.09)
-Theory of Mind animations (0-4)	3.61 (0.65)	3.57 (0.65)	0.123	-0.06 (-0.16;0.04)
-Goal-directed animations (0-4)	2.78 (0.85)	2.80 (0.83)	0.768	0.02 (-0.08;0.12)
-Random animations (0-4)	3.69 (0.60)	3.69 (0.60)	0.950	0 (-0.1;0.1)
MCQ-feelings (0-8)	5.24 (1.62)	5.37 (1.0)	0.131	0.10 (-0.00;0.20)
**AT-verbal**				
Intentionality (0-20)				
Theory of Mind animations	14.73 (2.38)	13.99 (2.65)	0.000*	-0.29 (-0.39;-0.19)
Goal-directed animations	9.73 (1.44)	9.59 (1.59)	0.023*	-0.09 (-0.19;0.01)
Random animations	2.00 (1.72)	1.97 (1.85)	0.404	-0.02 (-0.12;0.08)
Appropriateness (0-12)				
Theory of Mind animations	6.99 (1.81)	6.71 (1.80)	0.001*	-0.16 (-0.26;-0.06)
Goal-directed animations	9.26 (1.48)	9.14 (1.44)	0.061	-0.08 (-0.18;0.02)
Random animations	10.06 (1.96)	10.02 (2.01)	0.907	-0.02 (-0.12;0.08)

Abbreviations: AT-MCQ: Animated Triangles Task – multiple choice questions; AT-verbal: Animated Triangles Task – verbal response; SD: standard deviation; CI: confidence interval
