# Supplementary material for: A psychometric investigation of the multiple-choice version of Animated Triangles Task to measure Theory of Mind in adolescence
Source: PLoS One. 2022 Mar 10;17(3):e0264319. doi: 10.1371/journal.pone.0264319 (PMC8912123; doi:10.1371/journal.pone.0264319)
Supplement: S3 Table — (PDF) [file pone.0264319.s003.pdf]

Table S3. Comparison of Animated Triangles scores according to quartiles of scale scores in the IQ-subtest Block Design administered at age 11.

	**BD scale-score <8**	**BD scale-score 8-10**	**BD scale-score 11-12**	**BD scale-score 13-17**	**p-value**
	**N=204**	**N=249**	**N=207**	**N=233**	
**AT-MCQ**					
MCQ-categorization (0-12)	9.67 (1.56)	10.10 (1.30)	10.32 (1.21)	10.26 (1.21)	<0.001
-Theory of Mind animations (0-4)	3.48 (0.75)	3.61 (0.62)	3.68 (0.53)	3.67 (0.57)	0.004
-Goal-directed animations (0-4)	2.63 (0.91)	2.84 (0.80)	2.86 (0.76)	2.79 (0.79)	0.017
-Random animations (0-4)	3.56 (0.74)	3.65 (0.66)	3.78 (0.54)	3.80 (0.47)	<0.001
MCQ-feelings (0-8)	5.06 (1.67)	5.44 (1.58)	5.36 (1.48)	5.62 (1.43)	0.002
**AT-verbal**					
Intentionality (0-20)					
Theory of Mind animations	13.68 (2.65)	14.18 (2.61)	14.57 (2.50)	14.81 (2.49)	<0.001
Goal-directed animations	9.74 (1.65)	9.60 (1.50)	9.65 (1.36)	9.52 (1.36)	0.481
Random animations	2.45 (2.09)	2.05 (1.81)	1.58 (1.66)	1.69 (1.55)	<0.001
Appropriateness (0-12)					
Theory of Mind animations	6.38 (1.74)	6.85 (1.77)	6.96 (1.62)	7.36 (1.89)	<0.001
Goal-directed animations	8.92 (1.69)	9.08 (1.60)	9.31 (1.26)	9.46 (1.20)	<0.001
Random animations	9.60 (2.18)	9.97 (1.99)	10.51 (1.60)	10.39 (1.62)	<0.001

Abbreviations: AT-MCQ: Animated Triangles Task – multiple choice questions; AT-verbal: Animated Triangles Task – verbal response; BD scale score: Block Design scale score; SD: standard deviation.
