# Supplementary material for: A psychometric investigation of the multiple-choice version of Animated Triangles Task to measure Theory of Mind in adolescence
Source: PLoS One. 2022 Mar 10;17(3):e0264319. doi: 10.1371/journal.pone.0264319 (PMC8912123; doi:10.1371/journal.pone.0264319)
Supplement: S4 Table — (PDF) [file pone.0264319.s004.pdf]

Table S4. Comparison of Animated Triangles scores stratified by gender for adolescents with and without autism spectrum disorder (ASD).

	**Non-ASD n=1492**			**ASD n=54**		
	**Boys n=664**	**Girls n=828**		**Boys n=31**	**Girls n=23**	
	**Mean (SD)**		**p-value**	**Mean (SD)**		**p-value**
**AT-MCQ**						
MCQ-categorization (0-12)	10.09 (1.4)	10.06 (1.4)	0.851	10.00 (1.4)	9.96 (1.1)	0.689
MCQ-feelings (0-8)	5.25 (1.6)	5.40 (1.5)	0.092	4.97 (1.8)	4.17 (2.1)	0.183
**AT-verbal**						
Intentionality (0-20)						
Theory of Mind animations	14.76 (2.4)	14.00 (2.6)	<0.001	14.06 (2.7)	13.39 (3.3)	0.555
Goal-directed animations	9.74 (1.4)	9.61 (1.6)	0.029	9.39 (1.7)	8.91 (1.3)	0.234
Random animations	1.99 (1.7)	1.96 (1.9)	0.363	2.19 (2.0)	2.32 (1.7)	0.457
Appropriateness (0-12)						
Theory of Mind animations	6.99 (1.8)	6.72 (1.8)	0.002	6.97 (1.9)	6.39 (2.2)	0.405
Goal-directed animations	9.28 (1.5)	9.16 (1.4)	0.059	8.90 (1.5)	8.39 (1.9)	0.426
Random animations	10.08 (1.9)	10.06 (2.0)	0.992	9.77 (2.2)	8.65 (3.3)	0.233

Abbreviations: AT-MCQ: Animated Triangles Task – multiple choice questions; AT-verbal: Animated Triangles Task – verbal response; SD: standard deviation
