# Supplementary material for: A psychometric investigation of the multiple-choice version of Animated Triangles Task to measure Theory of Mind in adolescence
Source: PLoS One. 2022 Mar 10;17(3):e0264319. doi: 10.1371/journal.pone.0264319 (PMC8912123; doi:10.1371/journal.pone.0264319)
Supplement: S5 Table — (PDF) [file pone.0264319.s005.pdf]

Table S5. Comparison of Animated Triangles stratified by low and high IQ for adolescents with and without autism spectrum disorder (ASD) reported for subjects with low and high IQ separately.

	**Non-ASD n=870**			**ASD n=23**		
	**Low IQ n=442**	**High IQ n=428**		**Low IQ n=11**	**High IQ n=12**	
	**Mean (SD)**		**p-value**	**Mean (SD)**		**p-value**
**AT-MCQ**						
MCQ-categorization (0-12)	9.90 (1.4)	10.29 (1.2)	<0.001	10.09 (0.7)	10.42 (0.8)	0.344
MCQ-feelings (0-8)	5.27 (1.6)	5.53 (1.4)	0.048	5.00 (1.7)	4.58 (2.2)	0.925
**AT-verbal**						
Intentionality (0-20)						
Theory of Mind animations	13.97 (2.6)	14.74 (2.5)	<0.001	13.36 (3.0)	13.42 (2.4)	0.975
Goal-directed animations	9.68 (1.6)	9.60 (1.4)	0.486	8.82 (1.3)	9.08 (1.1)	0.557
Random animations	2.23 (2.0)	1.64 (1.6)	<0.001	2.45 (1.9)	1.27 (1.1)	0.085
Appropriateness (0-12)						
Theory of Mind animations	6.63 (1.8)	7.18 (1.8)	<0.001	6.91 (2.1)	7.00 (1.9)	0.803
Goal-directed animations	9.03 (1.6)	9.40 (1.2)	0.002	7.91 (1.0)	8.92 (1.6)	0.035
Random animations	9.82 (2.1)	10.47 (1.6)	<0.001	9.36 (2.2)	9.58 (2.9)	0.443

Abbreviations: AT-MCQ: Animated Triangles Task – multiple choice questions; AT-verbal: Animated Triangles Task – verbal response; SD: standard deviation
