# Supplementary material for: A psychometric investigation of the multiple-choice version of Animated Triangles Task to measure Theory of Mind in adolescence
Source: PLoS One. 2022 Mar 10;17(3):e0264319. doi: 10.1371/journal.pone.0264319 (PMC8912123; doi:10.1371/journal.pone.0264319)
Supplement: S6 Table — (PDF) [file pone.0264319.s006.pdf]

Table S6. Skewness and kurtosis for Animated Triangles scores.

	**Skewness**	**Kurtosis**
**AT-MCQ**		
MCQ-categorization (0-12)	-0.92	3.95
-Theory of Mind animations (0-4)	-1.72	6.60
-Goal-directed animations (0-4)	-0.55	3.20
-Random animations (0-4)	-2.12	7.87
MCQ-feelings (0-8)	-0.54	2.97
**AT-verbal**		
Intentionality (0-20)		
Theory of Mind animations	-0.41	2.53
Goal-directed animations	0.53	3.20
Random animations	0.98	4.30
Appropriateness (0-12)		
Theory of Mind animations	0.31	3.16
Goal-directed animations	-1.29	7.36
Random animations	-1.39	5.84

Abbreviations: AT-MCQ: Animated Triangles Task – multiple choice questions; AT-verbal: Animated Triangles Task – verbal response
